# Temporal expression patterns of *Pasteuria* spp. sporulation genes

**DOI:** 10.21307/jofnem-2019-039

**Published:** 2019-07-29

**Authors:** Ruhiyyih Dyrdahl-Young, Weiming Hu, Peter DiGennaro

**Affiliations:** 1 Department of Entomology and Nematology, University of Florida, Gainesville, FL 32611

**Keywords:** Biological control, *Pasteuria* spp, *Meloidogyne* spp, RT-qPCR

## Abstract

Endospore-forming bacterium in the genus *Pasteuria* spp. infect multiple agriculturally significant plant parasitic nematodes and has potential as a potent biological control. Success as a biological control requires not only spore attachment to the cuticle, but sporulation and reproduction within the nematode host. Tracking and identifying *Pasteuria* spp. development is then critical to demonstrating efficacy as a biocontrol. Microscopic observations suggest *Pasteuria* spp. follows the model bacterium, *Bacillus subtilis*, sporulation. Here, we identified *B. subtilis* homologs of sporulation regulators in *Pasteuria* spp. and characterized the temporal expression of these genes throughout the bacterium’s ∼30-d lifecycle in *Meloidogyne arenaria* as a means of tracking sporulation development. Detectable levels of transcripts of *Spo0F* were present as early as 5 d after the nematodes were exposes to *Pasteuria* spp. and were relatively constant throughout the 30-d lifecycle. Transcripts to *Sigma-F* were significantly higher in the middle of the lifecycle, while the transcripts of *Sigma-G* were detectable between 15 and 25 d, nearing the end of the lifecycle. These three markers can be used to track the process of sporulation in the nematode and augment microscopic observations. Tracking sporulation of *Pasteuria* spp. is important to fully realize its potential as a biological control method as it can more readily identify successful parasitism, define host ranges, and inform in vitro growth progress.

Some of the obligate, endoparasitic, spore-forming species of the bacteria *Pasteuria* spp. are antagonistic to plant parasitic nematodes. *Pasteuria* spp. is highly host specific; not all isolates of a given species will adhere to all phytoparasitic nematodes, and not all the endospores that adhere will germinate and infect the nematode (Mohan et al., 2012). Host specificity remains an essential barriers in deploying *Pasteuria penetrans* as a successful biological agent to combat agriculturally devastating, polyphagous root-knot nematode *Meloidogyne* spp. (Srivastava et al., 2018). The endospores that do successfully germinate, grow vegetatively and sporulate in the bodies of their host (Opperman et al., 2011), significantly reducing nematode fecundity as the multiplication of endospores in the body of infected females occludes the growth and development of eggs.

*Bacillus subtilis* is the traditional model to study the process of sporulation (Stragier and Losick, 1996). This process can be simplified into four stages: initiation, asymmetrical division, engulfment, and maturation. Bacterial sporulation is often triggered by environmental factors including insufficient carbon (Schaeffer et al., 1960) or nitrogen, damage or synthesis of DNA (Piggot and Hilbert, 2004), and cell density (Grossman, 1995). The environmental factors initiating sporulation in *P. penetrans* have yet to be elucidated. In *Bacillus*, initiation of the sporulation pathway begins with the phosphorylation of five histidine kinases. The transcripts of the early-stage sporulation genes *Spo0F*, *Spo0B*, and *Spo0A* (Parashar et al., 2011) are autophosphorylated, which triggers the transcription of genes controlled by the *Spo0A* regulon. In the second stage of sporulation, asymmetrical cell division, the vegetative cell is divided into the larger mother cell and a relatively smaller forespore (Piggot and Hilbert, 2004). This process is guided by *Sigma-E* in the mother cell and *Sigma-F* in the forespore. During the third stage of sporulation the mother cell engulfs the forespore (Fujita and Losick, 2003). In the final stage of sporulation, the mother cell is lysed (Hosoya et al., 2007), releasing the mature endospore. Several *B. subtilis* homologous genes involved in sporulation have been characterized in *P. penetrans*, namely *Spo0F*, *Spo0A*, and *SpoIIAB* (Preston et al., 2003; Schmidt et al., 2004; Trotter and Bishop, 2003) and four transcription factors (sigma factors: *Sigma-E*, *Sigma-F*, *Sigma-G*, and *Sigma-K*) in. This is the first report of nematode-parasitic *Pasteuria* spp. sporulation genes expression.

Utilization of *Pasteuria* spp. as a biological control against plant-parasitic nematodes is a promising supplement to conventional methods (Luc et al., 2010; Schmidt et al., 2010; Kokalis-Burelle, 2015; Baidoo et al., 2017). Microscopic observations have been used to describe the development of *P. penetrans* inside the body of its host nematode (Phani and Rao, 2018), but more accurate methods with higher throughput are, in the current study, transcripts of *Spo0F*, and the Sigma factors *Sigma-F* and *Sigma-G* are used as landmarks to track the development of *P. penetrans*, inside their respective nematode hosts. Changes in the expression of the genes involved in sporulation provide a degree of analytical precision that can augment microscopic observations.

## Materials and methods

### Plant and nematode culture and inoculation

Cowpea (*Vigna unguiculate)* seeds (‘Whippoorwill’ Baker Creek Heirloom Seed Co., MO) were used as the nematode host and germinated after sterilization and placed on moist paper until radical emergence. Seedlings were then transplanted into CYG Germination Pouches (Mega International, Newport, MN) for nematode inoculation. All plants were grown in individual pouches. Pouches were watered every 24 hr throughout the 30-d experiment with sterilized deionized water.

Freshly hatched *Meloidogyne arenaria* second-stage juveniles (J2) were exposed to *P. penetrans* at a rate of 1 J2: 200 endospores. The concentration of endospores was determined using a hemocytometer. Nematodes and endospore suspensions were centrifuged for 4 min at 1,300 *rcf.* At least 20 nematodes were subsampled and observed on an inverted microscope to ensure J2 had an average of at least five endospores attached to a nematode cuticle. Endospore encumbered nematodes were inoculated onto 8-d-old cow pea plants at the rate of 250 J2 per plant. After 48 hr, marking the beginning of the experimental period, plant roots were moved to fresh pouches to synchronize nematode penetration and prevent subsequent root penetration. Pouches were grown in growth chambers at a constant 28°C with 16 hr days.

Infected plant roots were collected at 0 and 5, 10, 15, 20, 25, and 30 d after infested roots were transferred to fresh pouches. Each plant root system was collected and frozen in liquid nitrogen in separate 1.5-ml microcentrifuge tubes, representing independent biological replicates.

### RNA isolation

Frozen root samples were masticated with a mortar and pestle with 2-ml of TRIzol (Invitrogen, Waltham, MA). RNA extraction was performed as per the manufacturer’s instructions. Purified RNA from infested plant roots was synthesized into cDNA using the iScript cDNA Synthesis kit (Bio-Rad, Hercules, CA). Each independent replicate for the seven times points evaluated was replicated in three 10 µl qPCR reactions using iTaq^TM^ Universal SYBR^®^ green Supermix (Bio-Rad, Hercules, CA). The qPCR experiments were performed using the ABI 7500 Real-Time Thermal Cycler (Applied Biosystems, Foster City, CA). The qPCR conditions cycle is as follows: 95°C for 20 sec, followed by 40 cycles of 95°C for 3 sec and 60°C for 30 sec. Melt curves of the amplicons are constructed using a temperature of 95°C for 15 sec and 60°C for 1 min. Standard curves were used to determine the copy number of each transcript. To construct each standard curve, a PCR was performed for each of the sporulation genes. The resulting PCR fragments were serially diluted ranging from 1e−4 ng/µl to 1e−2 ng/µl. These concentrations were converted into copy numbers of each gene of interest based on amplicon sequence and length. The standard curve was built by log transforming the DNA concentrations and fitting an exponential curve. Copy numbers for each gene of interest (*Spo0F*), Sigma-Factor (Sigma-*F* and Sigma-*G*) were quantified for each replicate based on *C*_*t*_ values and the standard curve.

### DNA sequencing

Genomic DNA was extracted and amplified from endospore populations as described in Mauchline et al., 2011. *Pasteuria* spp. sequences used for primer design were obtained from NCBI and using Geneious (version 10.1.3, Kearse et al., 2012). PCR products were sequenced (Eurofins Genomics; Louisville, KY) and validated against publicly available *Pasteuria* spp. sequences. The sequences of *Spo0F*, *Sigma-G*, and *Sigma-F* were used to build qPCR primers using Geneious^®^, primer builder was used to construct qPCR primers.

### Microscopy

At least 10 individual nematodes were excised from the roots of plants at each time point (0 hr, 5 hr, 10 hr, 15 hr, 20 hr, 25 hr, and 3 d) and water-mounted onto slides. When *Pasteuria* spp. structures were detected, photographs were taken using Zeiss Axio Vert A1.

### Data analysis

Three copy numbers were calculated for each of three replicates from all seven timepoints. To quantify changes in the expression level of *Spo0F*, *Sigma-F*, and *Sigma-G,* pairwise comparisons of each timepoint were conducted using a *t-*test (*α* = 0.05).

Five replicates from four timepoints were counted to quantify the numbers of sporulation structures present at that time point. Each picture was counted by two observers and an average of their counts was used for analysis. The timepoints analyzed were, 15, 20, 25, and 30 d after the nematodes were exposed to *P. penetrans.* To calculate a percentage of each sporulation structure, the number of each structure was divided by the total number of all the structures observed in the photograph and the multiplied by 100%. Pairwise comparisons using a *t*-test were conducted to detect statistically significant differences in the percentage of given sporulation structures across the four timepoints.

## Results

### Homologous sporulation genes

**Figure 1:**
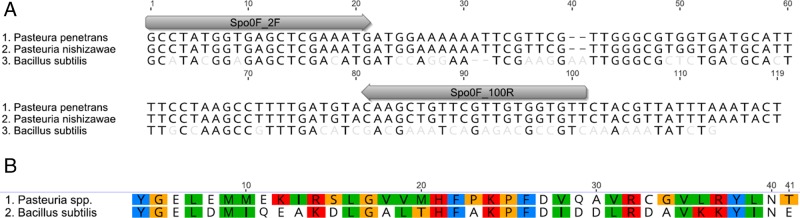
The *Bacillus subtillus* homologous sporulation gene *Spo0F* was identified and sequenced in *Pasteuria penetrans. Spo0F* nucleotide and translated amino acid sequence (A). The *Pasteuria* spp. sequences are identical and share a 72% identity with the *B. subtilis* sequence (B). The sequences and positions of primers used in the qPCR gene expression analysis for this gene are noted.

**Figure 2:**
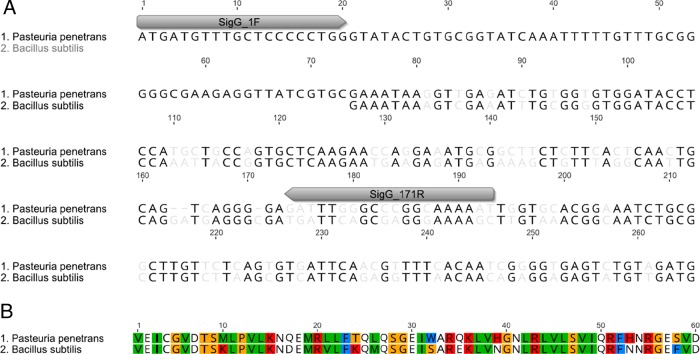
The conserved RNA sigma factor *Sigma-G* was identified in *Pasteuria penetrans.* The nucleotide sequence is 71% similar the *Bacillus subtilis* sequence (A). The amino acid sequence (B) has 83% identity with the *B. subtilis*, *Sigma-G.*

**Figure 3:**
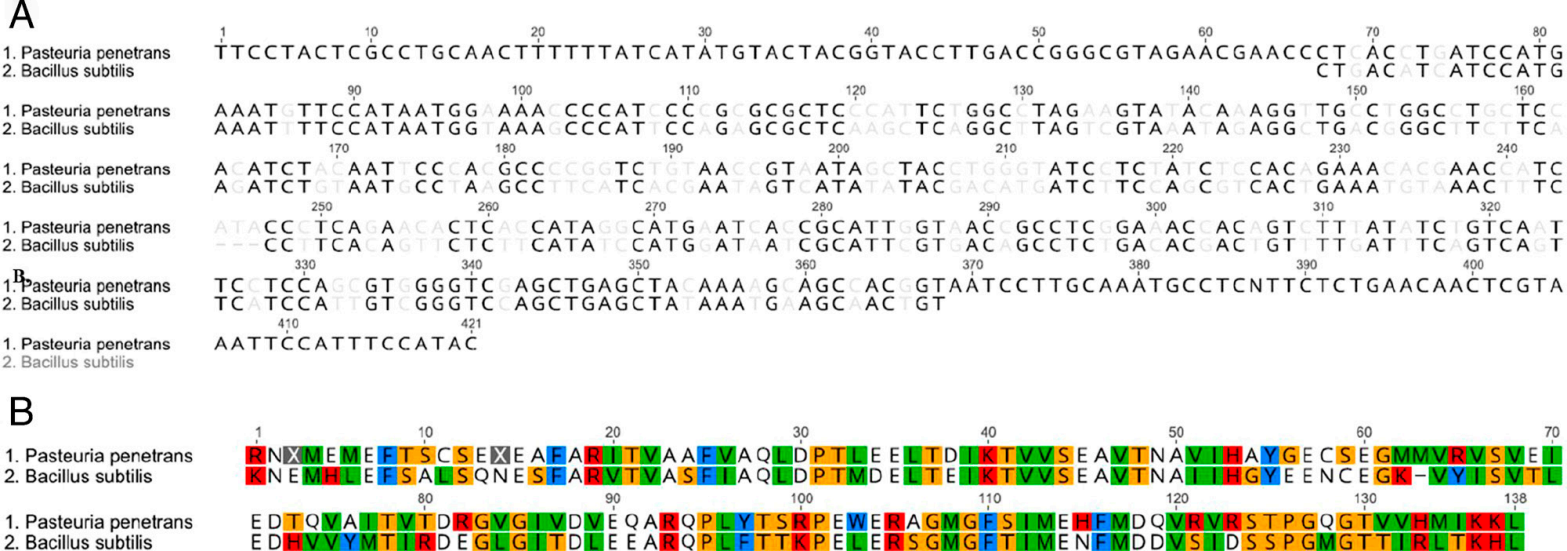
Nucleotide sequence (A) and amino acid residue (B) of *Bacillus subtilis* and *Pasteuria penetrans* sigma factor, *Sigma-F*.

Spo0F, Sigma-G, *and* Sigma-F: the process of sporulation is conserved among endospore forming bacterium. We sought to identify the homologous genes regulating sporulation in *Pasteuria* spp. Using reciprocal BLAST searches and *B. subtillis* sequences as query, we identified *Spo0F*, *Sigma-G*, and *Sigma-F* homologs within publicly available *Pasteuria* spp. sequences. The *P. penetrans* sequences of *Spo0F* identified and generated from endospore DNA here share a 100% identity with one another and a 58.4% identity with *B. subtilis* at the nucleotide level (Fig. 1), while the *Sigma-G* and *Sigma-F* sequences have a 70.7% and 66.9% identity with the corresponding *B. subtilis* gene (Figs. 2 and 3). The corresponding amino acid sequences for Spo0F, Sigma-G, and Sigma-F share a 48.8%, 83.0%, and 80.0% identity with the *B. subtilis* amino acid sequences, respectively (Fig. 1-3).

### Temporal gene expression

**Figure 4:**
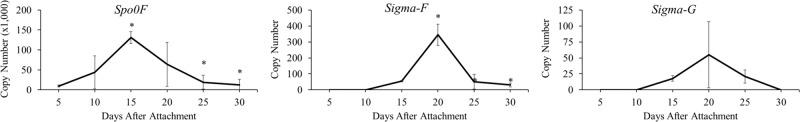
To track the lifecycle of the obligate endoparasite *Pasteuria penetrans,* the timing of the expression of *Bacillus subtilis* homologous sporulation gene *Spo0F* (A) and sigma factors *Sigma-F* (B) and *Sigma-G* (C) was quantified throughout the 30 d lifecycle using RT-qPCR. The results represent three independent replicates each from individual root systems. Pairwise comparisons using Student’s *t*-test (*α* = 0.05) were used to examine differences in the expression levels at all timepoints. *Statistically differences. There were significantly more copies of the transcript *Spo0F* on day 15 than days 25 or 30. There were also significantly more Sigma-F transcripts on day 20 than on day 25 or 30.

In *B. subtilis,* the gene *Spo0F* is active during the initiation of sporulation. The sigma factors *Sigma-F* and *Sigma-G* are active near the middle and end of sporulation, respectively. Monitoring the progression of *Pasteuria* spp. sporulation can help inform development of this endospore as a biological control. Using RT-qPCR, we quantified sporulation gene expression throughout *P. penetrans* life cycle; *Spo0F* transcripts were present at five days after the nematodes were exposed to *P. penetrans* and continued to be transcribed throughout the 30-day lifecycle. Importantly, pairwise comparisons (*t*-test, *α* = 0.05) revealed significant differences between each time point. There were significantly more copies of *Spo0F* transcripts early in the life cycle, on day 15, than on day 25 and on day 30. The forespore gene regulator, *Sigma-F*, was not detected until day five and had a significantly higher number of transcripts on day 20 than on day 25 and 30. Transcripts of *Sigma-G*, the late-stage sporulation regulator was only detected between 15 and 25 d (Fig. 4) but were not significantly different.

The nature of the expression patterns of *Sigma-G*, only being recorded at the later developmental stages, provides a second affirmation that the cDNA libraries had no genomic DNA contamination, such that amplification reported is only from RNA transcripts. The RNA from J2 exposed to *P. penetrans* was collected 48 hours after exposure (time point 0 d). Importantly, no transcripts of the three sporulation genes were detected at this time point. If gDNA contamination was present in the cDNA libraries, copies of the *Sigma-G* gene would have been detected at all time points (Fig. 4).

### Sporulation structure analysis

**Figure 5:**
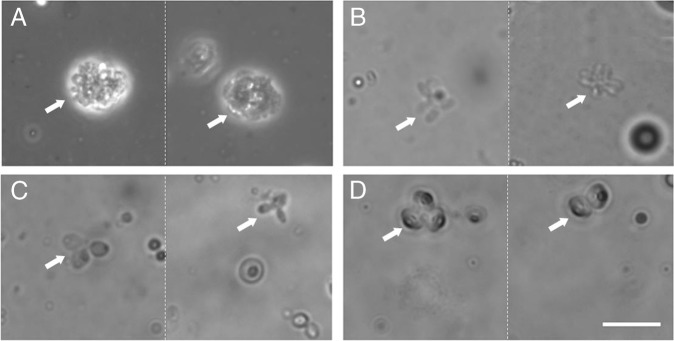
Light microscopic images of the development of *Pasteuria penetrans*. Photographs taken 10 (A), 15 (B), 20 (C), and 25 (D) days after *Meloidogyne arenaria* exposure to *P. penetrans.* Arrows indicate microcolonies (A), thali (B), nascent sporogonium (C) and sporogonium (D). Scale bar represents 10 µm, two images are shown for each developmental stage.

**Figure 6:**
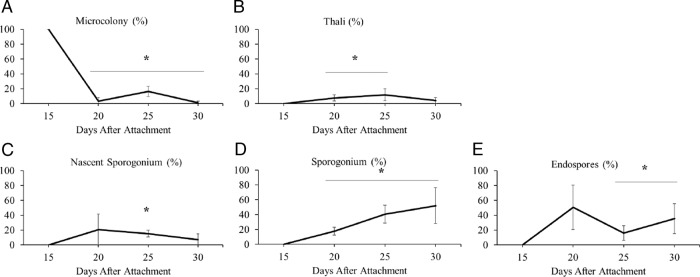
The percentage of each sporulation structures was assessed to track the lifecycle of *Pasteuria penetrans* in its nematode host. For each of the four time points assessed, five independent infected nematodes samples were excised from inoculated roots and the numbers of microcolonies (A), thali (B), nascent sporogonium (C), sporogonium (D), and mature endospores (E) were counted by two observers. Shown is mean observations expressed as a percentage of the total number of sporulation structures identified. Pairwise comparisons using *t*-test (*α* = 0.05) was conducted to compare a given time point to the initial time point (15 d).

To relate gene expression with traditional observations, we tracked the development of *Pasteuria* spp. sporulation structures using microscopy. Overall, the percentage of microcolonies, thali, nascent sporogonium, and mature endospores correspond with the gene expression analyses. Representative microscopic pictures are presented in Figure 5 for each of the notable sporulation stages. The percentage of microcolonies is significantly higher on day 15 than later in the lifecycle while the percentage of thali was higher between days 20 and 26 than on day 15 (Fig. 6). Sporogonium (both nascent and sporogonium) significantly more represented on day 25 than on day 15 (Fig. 6) and the percentage of endospores is highest in during the final days of observation 25 and 30 than on day 15 (Fig. 6).

## Discussion

Microscopy allows for the observation of endospores adhering to a cuticle of a nematode, however, the development of *Pasteuria* spp. inside of a nematode is difficult to assess for an entire population of nematodes using this method alone. Combining traditional observations with the expression of sporulation genes, it is possible to track the development of a population of the endoparasite *P. penetrans* inside its host nematodes as they are parasitizing root tissue. Expression of the sporulation genes enables assessment of an entire population of *Pasteuria* spp., whereas, microscopic observation only allows for an assessment of sporulation in individual nematodes. The transcription of *Spo0F* can serve as a marker for the initiation and progression of sporulation in *P. penetrans.* In the model organism *B. subtilis*, the protein encoded by this gene is a part of the phosphorelay system that controls the initiation of sporulation (Parashar et al., 2011). The expression of *Spo0F* throughout the sporulating bacterium’s lifecycle make this gene a potential positive control to indicate the presence and quality of RNA within a sample. The early expression of the *Spo0F* indicates the process of sporulation begins as early as 2 d after nematodes are exposed to a suitable host. The transcription of the *Spo0F* gene can possibly be used as a marker to assess if a given isolate of *P. penetrans* is virulent against a given population of nematodes. If the nematodes were exposed to *P. penetrans* and there was no transcription of the *Spo0F* gene, this would indicate endospores may have attached but did not infect the nematode and initiate sporulation.

The virulence of different isolates of *P. penetrans* against different populations of *Meloidogyne* spp. could be compared using *Spo0F* and *Sigma-G.* Transcripts of *Spo0F* would indicate how quickly sporulation is initiated inside the nematode while transcripts of *Sigma-G* would correspond to how rapidly *P. penetrans* endospores mature and fill the ovarian tissue of the nematode before the production of nematode eggs. This timing is key to assessing if *P. penetrans* infection will suppress the reproduction of the nematode population.

The *Sigma-F* factor is active in the forespore after segregation of the forespore and mother cell (Fimlaid and Shen, 2015). The sporulation structures present after this septation are referred to as thali (Fig. 1). In *P. penetrans* our analyses of spore structures across time points show the consistent presence of thali from day 20 to the end of the observation period and formation of mature endospores. There is an overlap in the presence of the sporulation structures as it is common to see a mix of developmental sporulation structures at a given time point. The overlapping expression of the *Spo0F* gene and *Sigma-F* confirm the asynchronous nature of the sporulation of *P. penetrans* (Atibalentja et al., 2004), even with the synchronized nematode inoculation of plant hosts. It is possible that not all the sporulation structures produce will complete the process of sporulation. There are significantly more transcripts of the *Sigma-F* on day 20 than on day 30, indicating significantly more of the *P. penetrans* are in the middle of their life cycle at day 20 than at day 30. This makes *Sigma-F* a possible indicator of the asymmetric division stage of the *P. penetrans* lifecycle.

Nematodes had a 48-hr window in which to penetrate root tissue. Nematodes that failed to penetrate during this window were removed and excluded from the analysis through the transfer of infected plants to new, sterile pouches. There are likely multiple genotypes present in the spore line used, all of which may complete their lifecycle at different rates. This heterogeneity would be an advantage to the parasite as multiple genotypes successfully colonizing and maintaining diversity within the reservoir of spores in the soil. In this way *Pasteuria* spp. overcomes a limitation of host-specificity. Maintaining an heterogenous population of *Pasteuria* spores in a field setting may allow increased parasite fitness and efficacy as a biocontrol agent.

In *Bacillus* spp. and other endospore formers, *Sigma-G* is active late in sporulation. It is expressed during the process of spore core and spore coat formation, and the formation of inner and outer forespore membranes (Fimlaid and Shen, 2015). We characterized *Sigma-G* as a maker to identify the final stage of sporulation and production of mature endospores. The expression between days 15 and 25 in *P. penetrans* make it an ideal marker to signify the initiation of the final phase of sporulation. This finding is also supported in our morphological analyses of sporulation structures (Fig. 6E).

The identification of these markers (beginning (*Spo0F*), middle (*Sigma-*F), and end (*Sigma-G*) of sporulation), allow for the monitoring of *Pasteuria* spp. development on a population scale. Differences in the timing of sporulation progression may inform variations in virulence of different isolates against different nematode populations and genotypes. Our developed RT-qPCR assessment of the gene expression patterns can supplement the sometimes subjective microscopic reports of a given sporulation structure with higher throughput.
